# Comparison of AIMS65, Glasgow–Blatchford and Rockall scoring approaches in predicting the risk of in-hospital death among emergency hospitalized patients with upper gastrointestinal bleeding: a retrospective observational study in Nanjing, China

**DOI:** 10.1186/s12876-018-0828-5

**Published:** 2018-06-28

**Authors:** Lei Gu, Fei Xu, Jie Yuan

**Affiliations:** 1 Department of Gastroenterology, Nanjing First Hospital, Nanjing Medical University, 68, Changle Road, Nanjing, 210006 China; 2 Nanjing Municipal Center for Disease Control and Prevention, Nanjing, China; 3 The School of Public Health, Nanjing Medical University, Nanjing, China

**Keywords:** Upper gastrointestinal bleeding, Rockall score, Glasgow-Blatchford score, AIMS65 score, In-hospital death, China

## Abstract

**Background:**

This study aims to compare the performance of AIMS65, Glasgow–Blatchford (GBS) and Rockall scores (RS) in predicting the death risk among emergency-hospitalized patients with upper gastrointestinal bleeding (UGIB) in regional China.

**Methods:**

A retrospective study was implemented between January 2014 and December 2015. Eligible participants were those who were hospitalized with UGIB. The outcome variable was in-hospital death, while explanatory variables were AIMS65, GBS and RS scores. Odds ratios (OR) and 95% confidence interval (CI) were estimated to assess the association of AIMS65, GBS and RS with death risk using multivariate logistic regression models. The areas under the receiver operating characteristics curve (AUC) of three scoring systems were computed to compare their predictive power.

**Results:**

Among 799 UGIB participants, 674 were non-variceal bleeding (NVUGIB) and 125 variceal bleeding (VUGIB) patients. AIMS65 (OR = 14.72, 95% CI = 6.48, 33.43) and RS (OR = 1.60, 95% CI = 1.20, 2.13) were positively associated with the risk of in-hospital death. Moreover, AIMS65 (AUC = 0.91, 95% CI = 0.84, 0.98) performed the best in predicting in-hospital death, followed by RS (AUC = 0.79, 95% CI = 0.72, 0.86) and GBS (AUC = 0.71, 95% CI = 0.59, 0.83) among overall UGIB participants. AIMS65 was also the best indicator to predict in-hospital death among either NVUGIB participants (AUC = 0.89, 95% CI = 0.80, 0.98) or VUGIB participants (AUC = 0.94, 95% CI = 0.89, 1.00).

**Conclusions:**

AIMS65, GBS and RS scoring approaches were all acceptable for predicting in-hospital death among UGIB patients irrespective of the subtype of UGIB in China. The AIMS65 might be the most powerful predictor.

## Background

Upper gastrointestinal bleeding (UGIB) refers to the acute bleeding caused by upper gastrointestinal tract lesions beyond the ligament of Treitz, including non-variceal upper gastrointestinal bleeding (NVUGIB) and variceal upper gastrointestinal bleeding (VUGIB) [1]. Over the past decades, the treatment and management of UGIB have been substantially improved, however the death rate among UGIB patients did not decline notably. The death rate was approximately 10% within overall UGIB patients and even as high as 35% among those with acute and chronic co-morbidities in Western countries [2–4]. In China, UGIB-specific death rate was estimated to be 4–14%, showing a heavy disease burden caused by UGIB [5].

The scenarios of prognosis are different for UGIB patients. Some patients with minor bleeding might get completely recovered without clinical treatment, while others with severe clinical symptoms might lose lives if they could not receive appropriate and effective clinical treatment timely. Therefore, it is of particular importance for clinical practitioners to effectively identify those UGIB patients who are at high risk of experiencing subsequent adverse outcomes. Recently, some scoring approaches have been developed to predict the subsequent outcomes for patients with UGIB, including Rockall score (RS), Glasgow Blatchford Score (GBS), Baylor Bleeding score, Cedars-Sinai Medical Center predictive index, Almela score and AIMS65 score. Among the above mentioned scoring approaches, RS and GBS are the most often-used [6–10]. However, they were not applied as widely as expected in clinical practice due to the complicated score calculation. Fortunately, the AIMS65 scoring system has recently been developed and validated to predict in-hospital death by Harvard Medical School in 2011 [11], which was more easily-calculated relative to either GBS or RS.

Considering that there are approximately 1 million UGIB patients visiting hospital each year and high mortality among these UGIB patients in China [5], it is of great significance to identify those UGIB patients who are at high risk of death (or other severe adverse outcomes) and provide them appropriate clinical treatment in time. Therefore, an effective and easily-used scoring approach is urgently needed for identifying those UGIB patients at high risk of death in China. Using hospital-based retrospective data, we aimed to evaluate the performance of AIMS65, GBS and RS scoring approaches in predicting the risk of death for hospitalized UGIB patients in China.

## Methods

### Participants

This was a retrospective hospital-based observational study. Participants were UGIB patients who visited the Department of Gastroenterology of Nanjing First Hospital with related signs and symptoms (i.e., melena, hematemesis, coffee ground vomiting, and/or abdominal pain) and were hospitalized for clinical treatment between January 2014 and December 2015. Those patients were excluded from the study if they (1) did not receive endoscopy examination as they had severe clinical symptoms and needed emergent clinical treatment to save their lives (*n* = 7), and/or (2) had missing data regarding calculation of GBS, RS and AIMS65. Finally, 799 of 814 UGIB patients were successfully included in this study. Prior to data collection, written informed consent was obtained from each alive participant or from his/her next of kin for the died. This study was approved by the Academic and Ethical Committee of Nanjing First Hospital.

### Data collection

For each participant, the following information were collected: age, gender, symptoms and signs on admission (including hematemesis, coffee ground vomiting, melena, syncope, mental status, blood pressure and pulse), co-morbidities (e.g., ischemic heart disease, diabetes mellitus, congestive cardiac failure, liver disease), profiles from laboratory tests (e.g., albumin level, urea, international normalized ratio, creatinine, hemoglobin), endoscopy examination records and subsequent clinical outcome events (in-hospital death or being alive).

### Study variables

#### Outcome variables

The primary outcome variable was in-hospital death (“Yes” or “No”), defined as any death occurred during the period of hospitalization due to UGIB attack.

#### Explanatory variables

Explanatory variables were GBS, RS and AIMS65 scores, which were used as continuous and categorical measures, separately, in our analysis. There were 8, 5 and 5 risk factors involved to compute the score of GBS, RS and AIMS65, respectively. Table 1 presented all the specific risk factors and scoring algorithms included in each scoring system.

**Table 1:** Factors and scoring algorithms included in GBS, RS and AIMS65

	Admission clinical factor	Parameter	Score
GBS	BUN (mmol/L)	6.5–7.9	2
		8.0–9.9	3
		10.0–24.9	4
		≥ 25.0	6
	Hemoglobin level (g/dL)	Male: ≥ 12 to < 13	1
		Female: ≥ 10 to < 12	1
		Male: ≥ 10 to < 12	3
		Male: < 10, female: < 10	6
	SBP (mm Hg)	≥ 100 to < 109	1
		≥ 90 to < 100	2
		< 90	3
	Other marker	HR ≥ 100 bpm	1
		Melena	1
		Syncope	2
		Hepatic disease or cardiac failure	2
RS	Age (yr)	< 60	0
		60–79	1
		≥ 80	2
	Shock	HR > 100 bpm	1
		SBP < 100 mmHg	2
	Comorbidity	IHD, CHF, any major comorbidity renal failure, liver failure	2
		metastatic malignancy	3
	Endoscopic finding	Mallory-Weiss tear or no lesion	0
		Peptic ulcer disease, erosive esophagitis	1
		Malignancy	2
	Stigmata of recent hemorrhage	Clean-based ulcer, flat pigmented spot	0
		Blood in upper gastrointestinal tract, clot, visible vessel, bleeding	2
AIMS65	Albumin (g/dL)	< 3.0	1
	INR	> 1.5	1
	Mental status	Altered	1
	SBP (mm Hg)	≤ 90	1
	Age (yr)	≥ 65	1

*GBS* glasgow-blatchford score, *BUN* blood urea nitrogen level, *SBP* systolic blood pressure, *HR* heart rate, *RS* rockall risk score, *IHD* ischemic heart disease, *CHF* congestive heart failure, *INR* international normalized ratio, AIMS65 AIMS65 score

### Statistical analysis

First, we conducted descriptive analysis using t-test (continuous variables) or Chi-square test (categorical variables). Then, using logistic regression models, we examined the relationship between outcome events and scores, separately. Two models were introduced: model 1 was a univariate analysis with each score as the single predictor; model 2 was a multivariate regression model with adjustment for participants’ age and/or gender and/or other potential clinical confounders (including albumin, urea, international normalized ratio, hemoglobin, blood pressure, pulse, comorbidity, mental status [12, 13], blood platelet count, prothrombin time or bleeding causes based on endoscopy exam) with consideration of specific variables included in each scoring system. Next, we used receiver operator characteristic curve (ROC) analysis, a widely used approach, to compare the predictive power of each scoring system on the risk of experiencing outcome events [14, 15]. ROC curve is a graphic representation of the relation between sensitivity and specificity for a diagnostic test. The areas under the receiver operating characteristics curve (AUC) were calculated with sensitivity as y-axis against 1-specifity as x-axis. With AUC, ROC curve can estimates and compares the predictive power of different tests or measures, which can assist with the choice of one test over the others. Generally, a perfect test will have an AUC of 1.0 and an AUC = 0.5 means the test performs no better than chance. Sensitivity and specificity of each scoring system were calculated at all possible cut-off points. The optimal cut-off value was identified based on the maximum sum of sensitivity and specificity for each scoring system. Data were double-entered and cleaned with EpiData 3.0 (The Epidata Association, Odense, Denmark) and analyzed using SPSS 21.0 (IBM Corp, Armonk, NY, USA).

## Results

### Selected participants’ characteristics

Among the total 814 patients with UGIB, 799 participants were eligibly included in this study, with a mean (SD) age of 57.46 (18.04) years and 612 (76.60%) of men. Table 2 displayed the selected characteristics for 799 participants in this study. There were only 15 patients (1.8%) excluded from our analysis due to incomplete information. No death case was observed among those 15 excluded patients. The 15 patients excluded from our analysis (mean ± SD: 73.93 ± 14.84, *p* < 0.05) were significantly older than those 799 participants included in this study. However there was no statistical difference in gender proportion between the 15 excluded (60% of men) and 799 included patients (76.60% of men, *p* = 0.13).

**Table 2:** Selected demographic and clinical characteristics of participants (*N* = 799)

Characteristics	*n* (%)
Men (%)	612 (77.22)
Age, yr. (mean ± SD)	57.46 ± 18.04
Clinical symptoms	
Melena	671 (84.0)
Coffee ground vomiting	90 (11.3)
Mental status or syncope	77 (9.6)
Signs and Laboratory Examinations (mean ± SD)	
Systolic blood pressure, mm Hg	118.37 ± 17.87
Pulse, bpm	85.17 ± 13.53
Hemoglobin, g/dL	9.48 ± 2.89
Urea, mmol/L	9.84 ± 5.50
Albumin, g/dL	3.37 ± 0.57
INR	1.10 ± 0.49
Comorbidities	
Ischemic heart disease	120 (15.0)
Diabetes mellitus	98 (12.3)
Congestive cardiac failure	25(3.1)
Liver disease	130 (16.3)
Liver failure	123 (15.4)
Chronic renal impairment	11 (1.4)
Endoscopic finding	
Mallory-Weiss tear	40 (5.0)
Peptic ulcer disease	484 (60.6)
Malignancy	65 (8.1)
Erosive esophagitis	9 (1.1)
Other diagnoses	201 (25.2)
Stigmata of recent hemorrhage	
Clean-based ulcer	462 (57.7)
Flat pigmented spot	279 (34.9)
Blood in upper gastrointestinal tract,	30 (3.8)
Clot	3(0.4)
Visible vessel	6(0.8)
Bleeding	19 (2.4)

Of those 799 UGIB patients, 125 (15.60%) were with variceal bleeding and 674 (84.40%) with nonvariceal bleeding. With respect to the causes of bleeding for these 799 patients, 484 (60.58%) participants were with peptic ulcer bleeding, 65 (8.14%) with cancer bleeding, 40 (5.01%) with Mallory-Weiss syndrome, 9 (1.13%) with erosive esophagitis and 201 (25.16%) with other diseases (e.g., gastro-oesophageal varices, acute gastric mucosal lesions, Dieulafoy’s lesion, or diverticular bleeding).

### Associations of RS, GBS and AIMS65 scores with the risk of in-hospital death

The death rate was 3.1% (25/799) among UGIB patients in this study. Table 3 presented the associations of RS, GBS and AIMS65 scores with the risk of in-hospital death among overall 799 participants. After adjustment for potential confounders, AIMS65 (OR = 14.72, 95% CI = 6.48, 33.43) and RS (OR = 1.60, 95% CI = 1.20, 2.13) scores were examined to be positively associated with the risk of death among the overall participants, while marginally significant link (OR = 1.09, 955CI = 0.93, 1.27) was observed between GBS score and death risk.

**Table 3:** The associations of RS, GBS and AIMS65 scores with the risk of in-hospital death among 799 UGIB participants in Nanjing, China

Scoring system	Death	*n* (%)	Score values (Mean ± SD)	Unadjusted odds ratio (95% CI)	Adjusted odds ratio (95% CI)
RS	No	774 (96.9)	2.86 ± 1.78	1	1
	Yes	25 (3.1)	4.88 ± 1.90	1.72 (1.38, 2.13)	1.60 (1.20, 2.13)^a^
GBS	No	774 (96.9)	8.31 ± 3.64	1	1
	Yes	25 (3.1)	11.24 ± 4.02	1.31 (1.14, 1.50)	1.09 (0.93, 1.27)^b^
AIMS65	No	774 (96.9)	0.69 ± 0.99	1	1
	Yes	25 (3.1)	2.00 ± 1.00	8.24(4.67, 14.54)	14.72 (6.48, 33.43)^c^

^a^RS: Adjusted for gender, Hb, Albumin, BUN, INR, Mental status/ Syncope, PT and PLT

^b^GBS: Adjusted for age, gender, Albumin, INR, PT, PLT and endoscopic findings

^c^AIMS65: Adjusted for gender, Hb, BUN, heart rate, co-morbidity, PT, PLT and endoscopic findings

Table 4 showed the associations of RS, GBS and AIMS65 scores with in-hospital death by type of UGIB. Among participants with NVUGIB, the scenarios of the associations of RS, GBS and AIMS65 scores with in-hospital death were similar to those within overall participants. However, for patients with VUGIB, only AIMS65 was examined to be positively associated with the likelihood of death.

**Table 4:** The associations of RS, GBS and AIMS65 scores with the risk of in-hospital death among participants by type of UGIB in Nanjing, China

Sub-group of participants	Scoring system	Mortality	*n* (%)	Score values (Mean ± SD)	Unadjusted odds ratio (95% CI)	Adjusted odds ratio (95% CI)
NVUGIB (*n* = 674)	RS	No	658(97.6)	2.27 ± 1.41	1	1
		Yes	16 (2.4)	3.94 ± 1.44	1.83 (1.38, 2.42)	1.79 (1.24, 2.58)^a^
	GBS	No	658(97.6)	7.82 ± 3.56	1	1
		Yes	16 (2.4)	10.06 ± 3.85	1.22 (1.04, 1.43)	0.99 (0.82, 1.18)^b^
	AIMS65	No	658(97.6)	0.58 ± 0.72	1	1
		Yes	16 (2.4)	2.31 ± 1.14	7.29(3.81, 13.94)	8.72 (3.54, 21.48)^c^
VUGIB (*n* = 125)	RS	No	116 (92.8)	2.86 ± 1.78	1	1
		Yes	9 (7.2)	4.88 ± 1.90	1.64 (0.88, 3.08)	1.95(0.79, 4.80)^a^
	GBS	No	116 (92.8)	8.31 ± 3.64	1	1
		Yes	9 (7.2)	11.24 ± 4.02	1.48 (1.07, 2.05)	1.26 (0.88, 1.82)^b^
	AIMS65	No	116 (92.8)	0.69 ± 0.99	1	1
		Yes	9 (7.2)	2.00 ± 1.00	28.88(3.76, 222.02)	244.11 (4.18, 14,266.12)^c^

^a^RS: Adjusted for gender, Hb, Albumin, BUN, INR, Mental status/Syncope, PT and PLT

^b^GBS: Adjusted for age, gender, Albumin, INR, PT, PLT and endoscopic findings

^c^AIMS65: Adjusted for gender, Hb, BUN, heart rate, co-morbidity, PT, PLT and endoscopic findings

### Predictive power of RS, GBS and AIMS65 scoring approaches on the risk of experiencing in-hospital death based on ROC analysis

**Fig. 1:**
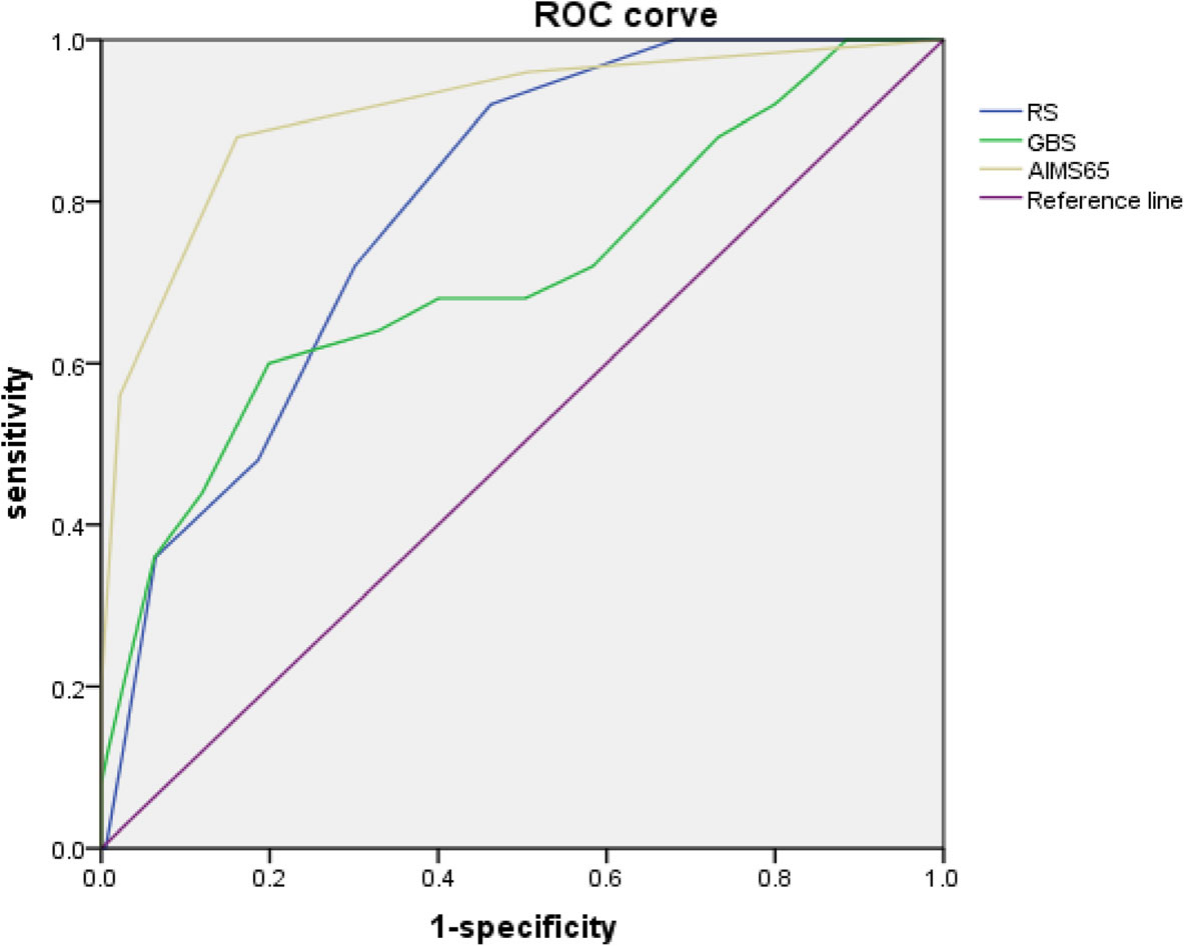
The receiver operating characteristic curves for the predictive value of AIMS65, RS and GBS for in-hospital death (Overall)

Figure 1 displayed the AUCs of RS, GBS and AIMS65 scoring systems to predict in-hospital death among overall participants. AIMS65 (AUC = 0.91, 95% CI = 0.84, 0.98) performed the best in predicting in-hospital death, followed by RS (AUC = 0.79, 95% CI = 0.72, 0.86) and GBS (AUC = 0.71, 95% CI = 0.59, 0.83).

**Fig. 2:**
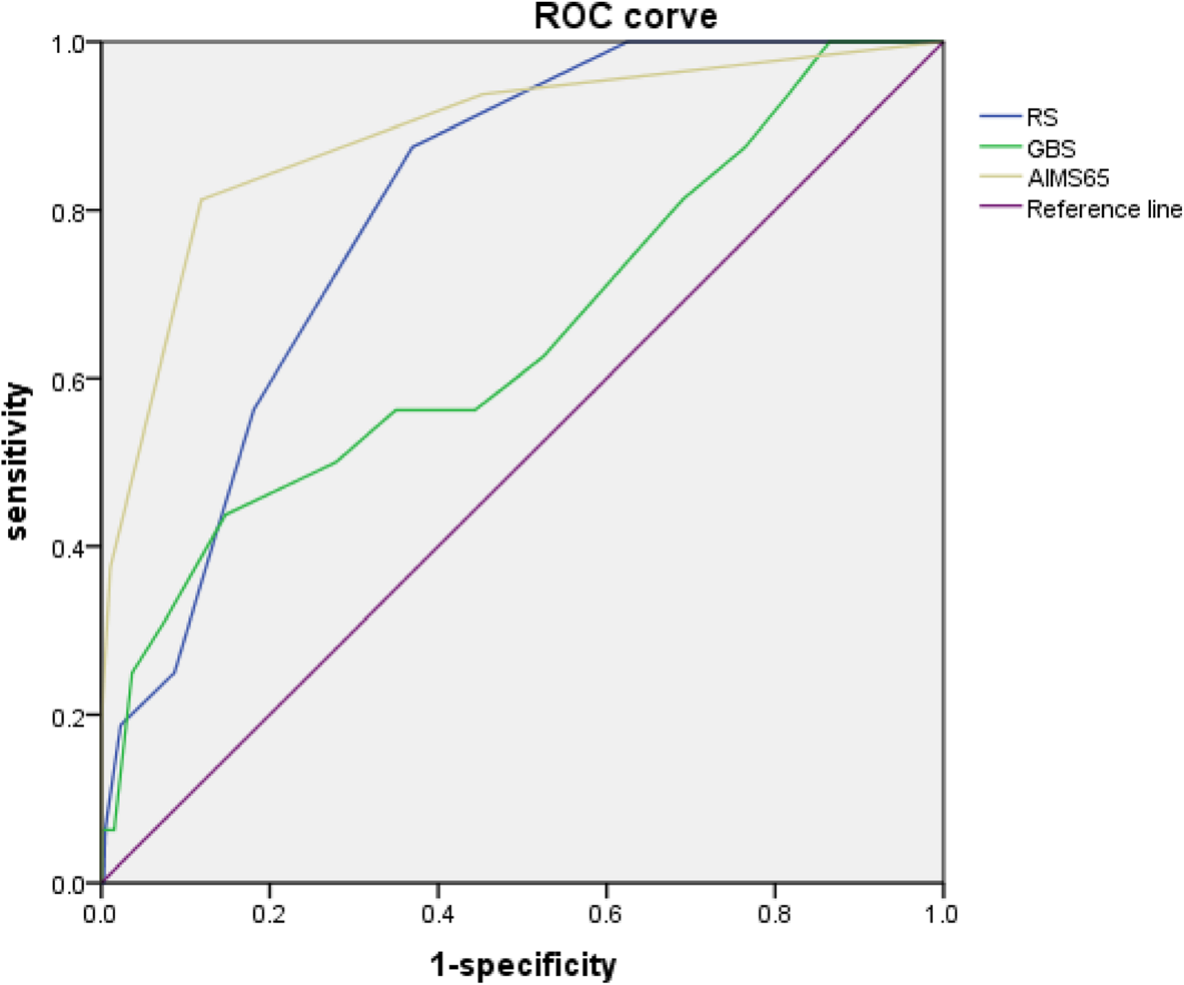
The receiver operating characteristic curves for the predictive value of AIMS65, RS and GBS for in-hospital death (NVUGIB)

**Fig. 3:**
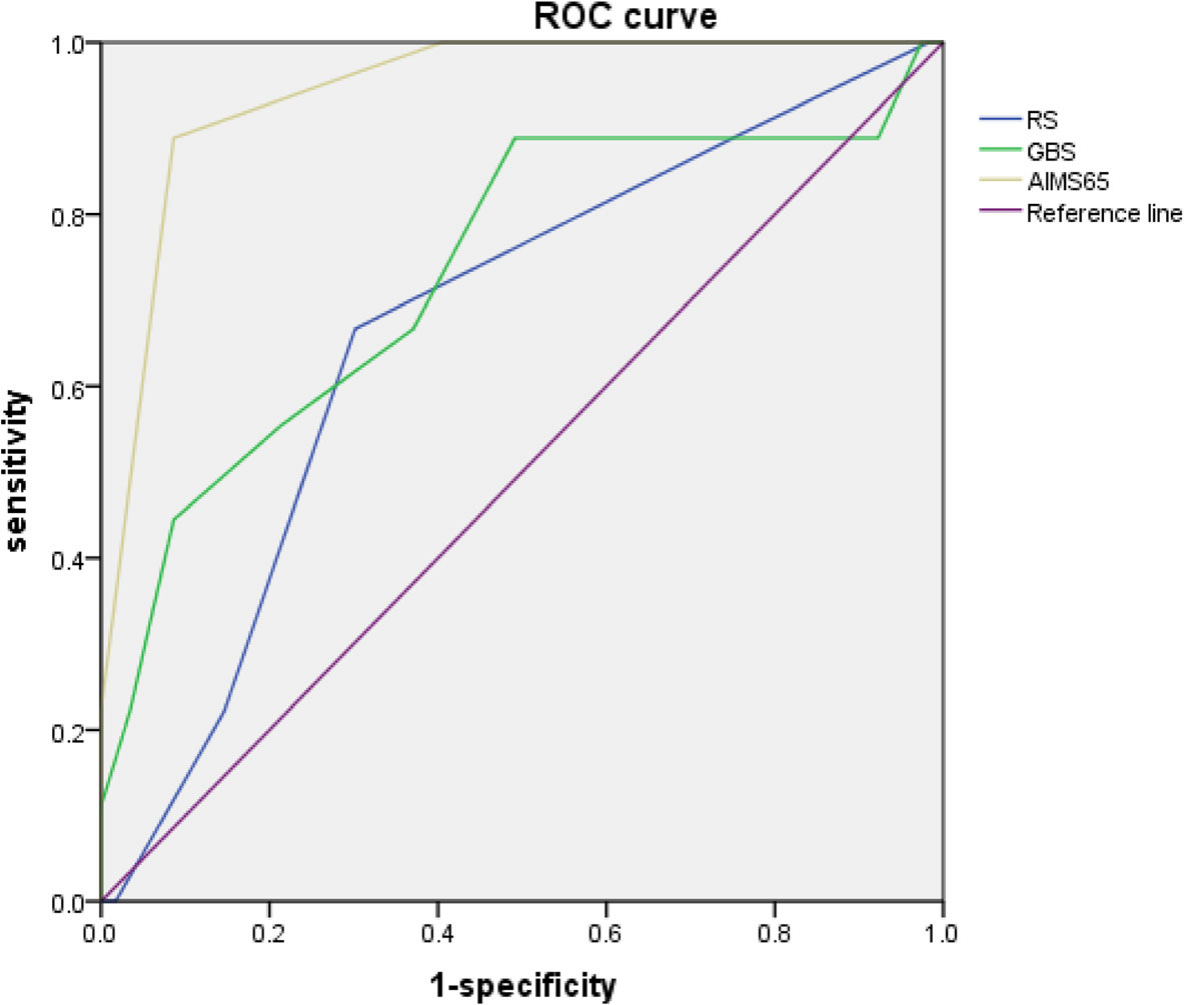
The receiver operating characteristic curves for the predictive value of AIMS65, RS and GBS for in-hospital death (VUGIB)

Figures 2 and 3 showed the AUCs of RS, GBS and AIMS65 scoring systems to predict in-hospital death among NVUGIB and VUGIB participants, separately. Among the NVUGIB participants, AIMS65 (AUC = 0.89, 95% CI = 0.80, 0.98) performed the best to predict in-hospital death, then RS (AUC = 0.81, 95% CI = 0.73, 0.88) and GBS (AUC = 0.65, 95% CI = 0.50, 0.80), while AIMS65 (AUC = 0.94, 95% CI = 0.89, 1.00) was also the best predictor of in-hospital death, and then GBS (AUC = 0.78, 95% CI = 0.54, 0.93) and RS (AUC = 0.67, 95% CI = 0.50, 0.84) among VUGIB participants.

### Optimal cutoff values of RS, GBS and AIMS65 scoring approaches for predicting in-hospital death

In this study, we estimated the optimal cutoff values of RS, GBS and AIMS65 scoring system, separately, for predicting in-hospital death among overall participants based on our ROC analysis (Table 5). The optimal cutoffs were identified to be 3, 12 and 2 for RS, GBS and AIMS65, respectively, and the largest sum of sensitivity (true positive rate) and specificity (true negative rate) produced for each scoring approach was 146% (RS), 140% (GBS) and 172% (AIMS65), separately, based on the corresponding estimated optimal cutoffs in this study.

**Table 5:** The estimated optimal cutoff values for AIMS65, GBS, and RS scoring system in this study

Scores	Cutoff value	Sensitivity	Specificity	Youden index
AIMS65	1	0.96	0.49	0.45
	2	0.88	0.84	0.72
	3	0.56	0.98	0.54
GBS	11	0.64	0.67	0.31
	12	0.60	0.80	0.40
	13	0.44	0.88	0.32
RS	2	1.0	0.32	0.32
	3	0.92	0.54	0.46
	4	0.72	0.70	0.42

## Discussion

In this retrospective study, significantly positive associations were identified between either RS or AIMS65 score and the risk of in-hospital death but marginally statistical link was observed for GBS scores among UGIB patients in Nanjing, China. Furthermore, based on AUCs, AIMS65 was the best approach, against the other two scoring systems, to predict in-hospital death (AIMS65 > RS > GBS for overall participants) among overall participants. Finding from this study suggested that these three scoring systems might be acceptable to predict in-hospital death for emergency hospitalized UGIB patients in China, which also added further evidence to existing literature.

The majority (91.7%) of the 799 UGIB patients were those with peptic ulcer bleeding, gastro-oesophageal varices, cancer bleeding, Mallory-Weiss syndrome or acute gastric mucosal lesions in our study. This was consistent not only with that reported from Western societies [9] but also with findings from a survey among Chinese UGIB patients [16].

The death rate (3.10%) observed among UGIB patients in this study was lower relative to those documented in previous literatures from both China and UK [17, 18]. This might be explained, in part, by that application of proton pump inhibitors (PPIs) prior to endoscopy, which could reduce the risk of re-bleeding and death [1]. In addition, different from some previous studies, the outcome events (in-hospital death) assessed in this study were limited to those observed in the period of hospitalization, therefore the potential death cases after discharge were not investigated, which might have the number of death cases underestimated.

Some recent studies found that AIMS65 had similar power to RS or GBS in predicting death for UGIB patients [5, 19]. However, our study showed that AIMS65 was better than either GBS or RS in predicting in-hospital death. In addition to the interesting findings for overall participants, our study demonstrated that each of AIMS65, GBS and RS also could be used to assess the risk of in-hospital death in either NVUGIB or VUGIB patients. This was in line with the reports from previous retrospective studies [20, 21].

Only five variables are included to compute the AIMS65 score and the calculation of AIMS65 score is really simpler relative to GBS or RS. In our study, AIMS65 performed the best among these three scoring systems to predict in-hospital death for UGIB patients. Therefore, in terms of easy-use and predictive power, AIMS65 might be the most optimal instrument for predicting in-hospital death among UGIB patients in China.

A cut-off value is critically important for each scoring system in predicting clinical outcome events. Unfortunately, cutoffs were almost different for each of these three scoring systems in previous studies [6, 19]. It is really difficult to explain such inconsistency of cutoff values among different studies. However, this might be partly due to some differences in those studies: participants and ethnicity, bleeding cause based on endoscopy exam, use of PPIs, time of endoscopy exam and adherence to guidelines regarding endoscopic therapy [19]. For example, a study from Spain found that the optimal cutoff value for predicting death among UGIB patients was 1 for AIMS65, 12 for GBS and 6 for RS [19]. An Australian study reported that the preferable cutoff was 3 for AIMS65, 15 for GBS and 7 for RS to predict death among UGIB patients [6]. And, our study suggested that the optimal cutoff value among Chinese UGIB patients was 2 for AIMS65, 12 for GBS and 3 for RS to predict in-hospital death. Therefore, the optimal cutoff of each scoring system should be specified for different population to maximize the power of identifying UGIB patients at high risk of death.

To the best of our knowledge, this is the first study conducted in China to compare the performance of GBS, RS and AIMS65 to predict the risk of in-hospital death among Chinese UGIB patients. The sample size was relatively large with sufficient number of VUGIB patients included, which allowed us to examine the predictive power of each scoring system within NVUGIB or VUGIB patients. Reasonable and appropriate statistical methods were used to investigate the association of scores generated by each instrument with the risk of death, to make comparison of predictive performance among the three scoring system, and to estimate the optimal cutoff for each scoring approach within this study.

Some limitations also need to be mentioned. First, this was a retrospective study, which implied potential information bias due to the study design, so the findings should be prudently interpreted. Second, 15 patients were excluded from our analysis due to incomplete data, which might cause potential bias in interpretation of the generalization of the present study findings. Third, only those UGIB patients died in hospital were included in this study and patients died after discharge were missed out. However, this might underpowered the effects of our analysis. In future, perspective studies are needed to further examine the performance of those scoring systems for predicting clinical outcome events among large scale representative sample population with UGIB in China.

## Conclusions

AIMS65, GBS and RS were all acceptable for predicting in-hospital death among either overall UGIB, NVUGIB or VUGIB patients in China. Among the three scoring systems, AIMS65 might be the best to predict in-hospital death for hospitalized UGIB patients.

## Abbreviations

AUC: Areas under the receiver operating characteristics curve
CI: Confidence interval
GBS: Glasgow–Blatchford
NVUGIB: Non-variceal upper gastrointestinal bleeding
OR: Odds ratios
ROC: Receiver operator characteristic curve
RS: Rockall scores
UGIB: Upper gastrointestinal bleeding
VUGIB: Variceal upper gastrointestinal bleeding
